# Changes in Segmental Body Composition in Children Practicing Martial Arts, Swimming and Team Sports

**DOI:** 10.3390/children13040482

**Published:** 2026-03-30

**Authors:** Anna Sojka, Bartosz Sojka, Agnieszka Chwałczyńska

**Affiliations:** 1 Doctoral School, Wroclaw University of Health and Sport Science, Al. IJ. Paderewskiego 35, 51-612 Wrocław, Poland; anna.sojka@awf.wroc.pl; 2 Faculty of Physiotherapy, Wroclaw University of Health and Sport Science, Al. IJ. Paderewskiego 35, 51-612 Wrocław, Poland; bartosz.sojka@o2.pl; 3 Department of Human Biology and Cosmetology, Wroclaw University of Health and Sport Science, Al. IJ. Paderewskiego 35, 51-612 Wrocław, Poland

**Keywords:** children’s body composition, BMI, taekwondo, rugby tag, swimming

## Abstract

**Highlights:**

**What are the main findings?**

- Supplementary physical activity introduced at an early school age has a positive impact on children’s physical fitness, weight, and body composition;
- Introducing diverse forms of physical activity into physical education classes has a positive impact on the willingness to participate in these classes and on the fitness, weight, and body composition of early school-age children.

**What are the implications of the main finding?**

- Introducing swimming, martial arts or team sports, which are less popular in a given area, to schools should make physical education classes more attractive and show children the role of training in improving their physical fitness.
- School-based PA alone is insufficient to meet WHO guidelines, highlighting the need for structured extracurricular programs.

**Abstract:**

**Background:** In the year 2022 more than 390 million kids and teenagers aged between 5 and 19 years old were overweight, including 160 million individuals who were suffering from obesity. It is commonly known that, in order to have a possitive change in physical and mental health, physical activity is needed. The aim of this study was to determine whether the type of physical activity undertaken influenced changes in body mass and physical fitness scores in children aged 7–10 years who practice taekwondo, swimming, and team sports, using tag rugby as an example. **Material and Methods:** A total of 200 children were eligible to participate in the research project and the analysis was conducted on 150 subjects (49% girls and 51% boys). Group IA (n = 20) trained in taekwondo, group IB (n = 42) trained in swimming, and group IC (n = 20) trained in rugby tag. Group II control subjects (n = 68) did not participate in any additional sports activities. Children’s body height was determined with a SECA 213 heightometer and body mass and body composition using Tanita’s MC-780 eight-electrode body composition analyser. Selected Eurofit tests were used to assess physical fitness. **Results:** A peripheral distribution of fat mass was found in all subjects, with the highest levels determined in the upper limbs. The highest values, apart from arm muscle strength, were found in the swimming group. Significant changes were observed in the subjects training in taekwondo. **Conclusions:** Results suggest that physical activity targeting the development of different motor skills should be part of preventive health care for children.

## 1. Introduction

According to data published by the World Health Organization (WHO), the number of obese adults has doubled since 1990 and even quadrupled among adolescents [1]. In Poland, the number of overweight people under 18 years of age is growing dynamically, from 6.5% of the population in 2010 to 13.4% in 2025 [2,3,4]. The occurrence of obesity in pre-pubertal children results in 90% of excess body weight in adulthood [5,6]. This is confirmed by research conducted by the National Childhood Measurement Programme, where, in the six-year observation period, an increase in the problem of overweight among 4–5-year-old children was observed from 10% to 21% [7]. Similar results were obtained by researchers assessing the problem of overweight and obesity in Poland and the United States [8,9]. It is widely known that physical activity is necessary to achieve positive changes in physical and mental health [10]. For the health and well-being of children and adolescents, the WHO recommends 60 min of moderate or intense aerobic physical activity per day [11]. Physical activity (PA) in the progressive age, along with the physiological increase in body weight, primarily affects the reduction in fat mass and the increase in muscle mass [12], thus improving physical fitness [13] and aerobic capacity [14,15], general health indicators [16,17,18], motor coordination, and the ability to learn and improve motor skills [19,20].

Basic motor skills such as strength, speed, endurance, and power, as well as secondary skills such as balance, flexibility, hand-eye coordination, and dexterity, develop from 4–5 years of age, but their peak occurs at different times [21]. In preschool and early school age, the emphasis is on the overall development of motor skills, with the introduction of activities that develop complex motor skills as children grow older [22,23,24,25]. Martial arts have been observed to have a beneficial effect on anthropometric values, including body mass and composition, particularly the distribution of fat and muscle mass, improved posture, and physical fitness, particularly motor coordination, balance, strength, and power [13,26,27,28]. The specific nature of water-based activities, a different environment from humans, positively impacts body composition, particularly the reduction in fat mass, and improves blood lipid levels, as well as cardiorespiratory fitness, thus contributing to overall physical fitness [29,30,31]. Rugby is another sport that has been shown to increase lean body mass and improve body composition [32]. Furthermore, tag rugby, a form of introductory rugby training, helps develop gross motor skills and overall fitness in children aged 5–6 [33].

Many authors focus on the need to incorporate physical activity to combat existing problems related to abnormal body weight or low physical fitness in school-aged children [5,8,10,12,13,14,16,17,25,28]. Therefore, it is crucial to determine whether forms of physical activity are a significant element in shaping motor skills and preventing overweight and obesity and whether and how individual forms influence body composition, particularly the development of muscle mass and the reduction or stabilization of fat mass.

Body mass assessment in younger school-age children is often based on medical checkups—health checkups. It is most often limited to body weight and height and the BMI (weight-height index) calculated from these. However, authors examining ontogenetic changes in body mass [6,8,10,13,15,21,22,24,26,27,29] emphasize not only the general concept of body mass but also the components of its composition. In adults, attention is paid to the distribution of fat mass, defining body types as android, where fat mass accumulates at the abdomen, and gynoid at the hips. In children, this distribution is not yet defined. Based on our own research and that of others, it has been observed that fat mass in children is distributed in a rather characteristic manner—peripherally [13,21,34]. The highest values of fat mass were observed in the upper limbs, particularly on the non-dominant side. When selecting physical activity for children aged 7–10, care should be taken to ensure that the activation involves both the dominant and non-dominant sides.

The aim of this study was to determine whether the type of physical activity undertaken influenced changes in body mass and physical fitness scores in children aged 7–10 years who practice taekwondo, swimming, and team sports, using tag rugby as an example.

By comparing three different forms of physical activity, it was assumed that each would significantly positively impact changes in body composition, with particular emphasis on reducing fat mass, compared to children who did not engage in additional physical activity. At the same time, it was assumed that the forms of physical activity undertaken would significantly impact motor skills such as balance, speed, abdominal muscle strength, and explosive power.

## 2. Materials and Methods

### 2.1. Consents

This study was conducted in accordance with the Declaration of Helsinki, and the research project was approved by the Senate Ethics Committee Wroclaw University of Health and Sport Science (No. 12/2019, approval date 15/03/2019). The study was conducted in accordance with the CONSORT statement (Consolidated Standards of Reporting Trials). The study was registered as part of a clinical trial under number NCT 06419829. Data obtained in accordance with the Personal Data Protection Act of 2018 (Journal of Laws of 2018, item 1000) were secured, and the children’s parents signed written consent to the use of data, including medical data, for the purposes of study recruitment (conditions for exclusion from the study group), the child’s participation in the project, and processing the results for scientific purposes and publication.

The sample size was determined based on analysis in the Sample Size Calculator, a test power of 0.8, an effect size of 0.5, and alpha < 0.05, which would result in a sample size of 161 participants. Randomly selected participants were: martial arts centers offering teak wood classes and a randomly selected trainer from a given martial arts school; swimming pools; and randomly selected swimming schools. Sports centers that met the eligibility criteria were randomly selected. The sports centers met the eligibility criteria: they were located in a large city, offered classes for children aged 6–10 as an introduction to the given sport, had more than one group at a given level, conducted classes in accordance with the guidelines of the individual sports federations, and had consent from the club’s management to conduct the research project. Randomization was supplemented by the random selection of a coach who conducted classes for the study group. According to the criteria, the coach had to hold instructor qualifications for the given sport issued by the relevant sports federation, have a clean criminal record, conduct classes with children aged 6–10, and have at least 10 years of training experience in the given sport. Inclusion and exclusion criteria were considered in the selection of participants for the groups based on questionnaires completed by parents.

One of fifty public primary schools offering classes for children in grades 1–3 that met the eligibility criteria was selected for tag rugby classes. Among the students whose parents consented to the extracurricular activities, the children were randomly assigned to the experimental and control groups on a 1:4 basis. After the experiment, the control group had the opportunity to participate in similar classes. Children participated voluntarily in each randomly selected training group.

The inclusion criteria for all subjects were written: informed consent of parents and informed consent of the child to participate in the study and research project, the child’s age being 7–10 years old, the absence of health contraindications to physical activity and the absence of metal elements in the body (screws, plates, anastomoses, etc.), and sexual development assessed on the Tanner scale at the level of M1 (girls), G1 (boys) and P1—childhood period (before puberty). For the children qualified for the study group, additionally, there was a lack of previous participation in relevant forms of physical activity and undertaking other, apart from the ones examined, organized forms of physical activity for more than once a week for 60 min (total extracurricular activities—3 × 60 min per week).

The criteria for exclusion from the research project were failure to meet at least one of the criteria listed above, active cancer, and voluntary resignation from the research project during its duration or lack of at least 85% training attendance for the study groups.

### 2.2. Study Group

**Figure 1:**
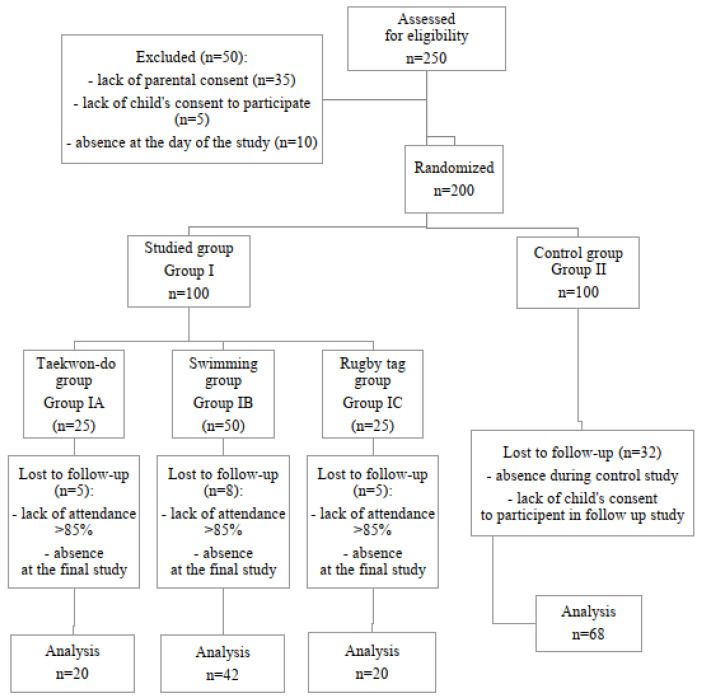
Consort diagram.

A total of 200 children were eligible to participate in the research project and the analysis was conducted on 150 subjects (49% girls). The mean age of the children studied was 7.63 years (Q1—7.28; Q3—8.23), mean body height 130.00 cm (Q1—125.0; Q3—135.0), mean body weight 28.5 kg (Q1—24.6; Q3—34.2), and mean BMI 17.2 kg/m2 (Q1—14.9; Q3—18.9). The children were divided into 2 groups: study group I (n = 82) and control group II (n = 68). Subjects in group I were randomly assigned to different forms of physical activity. Group IA (n = 20) trained taekwondo, group IB (n = 42) swimming, and group IC (n = 20) rugby tag. Participants in the control group II (n = 68) did not participate in any additional sports activities. The recruitment process for the study group is shown in the consort diagram (Figure 1).

### 2.3. Research Methodology

The research was carried out in multiple stages.

The parents of the studied children completed a personal questionnaire on personal data (social data (family size and parental employment), medical data (illnesses, injuries, and treatment) and physical activity data (Physical Activity Questionnaire—Children (PAQ-C)).

Stage I—Qualification for the research project

Children’s body height was determined with a SECA 213 heightometer and body mass and body composition using Tanita’s MC-780 eight-electrode body composition analyser. Total weight (BM), fat mass in percentage (FM%) and kg (FM), fat-free mass in kg (FFM), and predicted muscle mass (PMM) were used for analysis. Body composition was determined overall and by segment: right lower limb (RL), left lower limb (LL), right upper limb (RA), left upper limb (LA) and trunk (TR). BMI was calculated from height and weight.

Selected Eurofit tests were used to assess physical fitness: a balance test (standing on a horizontal bar with a wide stance), a strength test divided into explosive strength (jumping for a distance from a standing position), dynamic trunk strength (leaning forward while lying down), and static strength (gripping hands with maximum force on a dynamometer), power determined by upper limb muscular endurance (hanging on a horizontal bar), a flexibility test (leaning forward while sitting upright), and elements of eye–hand coordination, i.e., hand movement speed (alternately touching two appropriately spaced discs). The measurements were conducted quantitatively and compared with percentile values in individual trials, taking into account the age and gender of the participants. The scoring scale was 0–100 points, with 50 being the population average for age and gender.

Stage II—implementation of targeted physical activity

Each class began with a check of the attendance list to ensure the attendance requirement (85%) was met. Children attended classes in attire appropriate for the given sport: swimsuit, cap, goggles, kimono, and sportswear. Each activity was taught twice a week for 60 min, for at least 12 weeks, as an extracurricular activity. Training was conducted by qualified staff, using a methodology designed for beginners who had never played a given sport before.

Taekwondo classes (group IA)

Classes were held in a training room, lined with specialized mats for martial arts training. The room was equipped with professional punching bags, trowels and kicking discs, as well as wall ladders, mattresses, cones, coordination ladders and balls of various sizes. The classes were conducted according to the International Taekwondo Federation (ITF, https://itf-tkd.org/, 9 September 2025) guidelines. The introductory part included a warm-up in the form of a movement game and dynamic stretching. The main part was devoted to perfecting the technique, strength and dynamics of kicks and learning formal circuits. The final part again featured elements of play, as well as exercises leading to calming the body after physical exertion.

Swimming classes (group IB)

Classes were held in a 25-meter swimming pool (average water temperature: 28.8 degrees C; water depth 1.35 m–1.8 m) divided into 5 lanes. Classes were held according to the principles of learning to swim from scratch—familiarization with water, learning basic locomotory movements in water using swimming boards. The lessons included basic upper and lower limb movements for kink and backstroke, as well as jumping into the water from a standing position on the feet. Games using balls, weights retrieved from the bottom of the pool and foam mats for balance exercises—climbing onto the mat, running across the mat, and jumping into the water—were introduced as part of the warm-up and final section.

Rugby tag class (group IC)

The classes were held in a gymnasium equipped with mattresses, Swedish benches, wall-mounted ladders or cones, among other things. The classes introduced elements of rugby tag for the younger children, using balance, general fitness and agility exercises. Each class was tailored to the group and, although some exercises were repeated, the configuration was always different. An important part was the warm-up, during which the children had the chance to familiarize themselves with the rugby tag equipment, namely, Velcro straps, tags (ribbons attached to Velcro straps) and rugby balls, size 3–4 by Rhino and Kipsta. Exercises were chosen to be interesting for the children, innovate a new sport and include tasks to stimulate the lower limbs to work harder.

Stage III—execution of control tests

After completion of the 12-week training program, weight and body composition were reassessed using Tanita’s MC-780 eight-electrode analyser and physical fitness was assessed using the Eurofit test. To assess any changes in physical activity, the parents of the study children again completed the PAQ-C questionnaire. The mean time between stages I and III was 14.1 weeks.

Potential confounding factors

The project did not include dietary monitoring of participants; however, parents were asked not to make any dietary changes during this period. Monitoring the diet of a group of 7–10 year olds in their natural environment is very difficult. Even if we impose a diet, the child does not always realize the consequences of a single cookie, candy bar, or uneaten sandwich. Therefore, to minimize the error resulting from the lack of real dietary monitoring, a control group was introduced. This group did not participate in additional physical activity classes, attended schools in the same area, and the entire group was tested at the same time each year.

### 2.4. Statistical Analysis

The results were processed using the Statistica 13.3 package. The independent variables in this project are the types of sports or lack thereof. The dependent variables are body mass and composition and their changes, as well as the results obtained during tests of specific motor skills. Using the Shapiro–Wilk normality test, no normality of the distribution of the study variables was found. Frequency tables and descriptive statistics (median and Q1–Q3) were used to describe the group. The Wilcoxon non-parametric paired rank-order test was used to assess changes in the dependent groups. The non-parametric Kruskal–Wallis test was used to compare independent groups. Spearman’s rank correlation was used to assess relationships between variables. A statistical significance level of *p* < 0.05 was adopted.

## 3. Results

When comparing the anthropometric data of the subjects in each group, statistically significant differences were found in terms of age. The subjects in the IC group were the youngest and also had the lowest intra-group variation. The subjects in the control group were the oldest and had the highest body weight and BMI.

When analyzing body composition before the implementation of targeted physical activity, the highest fat mass was found in the control group. In the study group, in the majority of children, total fat mass was within the normal range for age and gender (for girls 15–25%; for boys 13–20%). A peripheral distribution of fat mass was found in all subjects, with the highest levels determined in the upper limbs. Statistically significant differences were found between the study and control groups. The results are shown in Table 1.

In all subjects, tests from the Eurofit test showed age- and gender-medium physical fitness. The highest values, apart from arm muscle strength (overhang on a bar), were found in subjects from group IB. Statistically significant differences in the results were observed for balance, flexibility, explosive strength and arm muscle strength tests. The results of the fitness tests are shown in Table 2.

After completing the targeted physical activity sessions, the anthropometric and fitness values of the studied children were re-evaluated. Only group IB observed a reduction in fat mass (positive results) after the training program. Statistically significant changes were noted in total fat mass (Group IC, *p* = 0.03), the right upper limb (Group IC, *p* = 0.004), the left upper limb (Group IB, *p* = 0.03; Group IC, *p* = 0.006), the right lower limb (Group II, *p* = 0.02), and the trunk (Group IC, *p* = 0.01).

When comparing fitness results, a negative difference is a positive result, as it indicates higher fitness after the targeted physical activity. As the results indicate, significant changes were observed in people practicing taekwondo (group IA). Detailed results are presented in Table 3.

Medium statistically significant positive correlations were observed between changes in age and BMI and balance. Low positive statistically significant correlations were observed between changes in left lower limb fat mass and explosive strength, flexibility, and speed of hand movements and upper limb muscular endurance. The results are shown in Table 4.

## 4. Discussion

The aim of the present study was to evaluate the effect of a three-month targeted physical activity program on body composition and physical fitness in children aged 7–10 years old. The results showed significant differences between the control group and the children studied, participating in sports activities (taekwondo, swimming, and rugby), in both anthropometric parameters and motor abilities. An interesting aspect is the comparison of basic anthropometric values in the study groups even before the intervention, i.e., in the preliminary study. Children undertaking physical activity in the project described here, who participated in taekwondo, swimming and tag rugby training, respectively, are statistically significantly lighter and, in fact, their BMI is lower than in children in the control group. This may indicate a lack of willingness on the part of children with elevated body weight to engage in targeted physical activity, which causes weight gain and puts them into a so-called vicious circle: lack of physical activity results in higher body weight, which, in turn, increases reluctance to engage in physical activity due to being overweight or obese. A way to avoid this situation could be to introduce more attractive forms of physical education within the time spent at school, minimize exemptions from physical activity classes or introduce additional forms aimed at weight reduction [34]. A similar relationship in the reduction in physical activity by children, which leads, among other things, to overweight or obesity, has been observed in studies by Kohl et al. [35], Bidzan-Bluma and Lipowska [36] or Soto-Lagos et al. [37] and emphasizes the strategic role of the school and the activities conducted there. In the described project, participation in the different training groups was voluntary. This means that, once a child had qualified for the study group, he or she could refuse to participate in the classes or terminate them at any time. The most common reasons for quitting were long-term illness or an inappropriate choice of sport. It is not a bad thing to not continue with a particular sport if children take up another form of physical activity. A child of younger school age has the right to seek out his or her interests. When choosing a sporting activity, a child is often guided by the opinion of its parents or peer group. However, this does not mean that he or she has an aptitude or desire for a particular sport.

In line with ontogenetic development, complex motor skills are refined and developed during the preschool and school years, requiring greater effort from the child. In the presented project, each of the training forms assessed required different skills from participants while simultaneously honing the motor skills necessary for their implementation. In swimming, a child must first acclimate to a different environment, namely water, and motor coordination develops through alternating movements. Balance, especially in a horizontal position, is also developed, as are strength and power through overcoming water resistance. In taekwondo, the greatest emphasis in initial training is placed on balance in various support positions on various surfaces (training mats)—standing on both legs, straddling, standing, on one leg; motor coordination—alternating movements; and strength and power—through swings and strikes. Introductory training to tag rugby is primarily based on developing speed and movement variability—running, starting, stopping, changing direction, coordination while working with the ball, and leg strength. The variability of training that accompanies the assessed disciplines often results in discontinuation of training and the search for a less demanding sport [24]. Improving motor skills requires time and consistent training. The 12-week period chosen for this project is the minimum time needed to achieve initial training results—passing the first taekwondo exam, familiarizing oneself with the water, and learning basic swimming strokes and basic ball handling techniques. Properly conducted training methods, repetitive movements, and learning proper technique and biomechanics of movement improve the motor skills essential for this sport. In this research project, a decrease in fat mass was observed in the group of children training in swimming after the intervention, with statistical significance noted only in the left upper limb. The statistically significant changes observed in the left upper limb may result from the activation of this segment. The children studied were right-handed, so all precision activities associated with fine motor skills, such as writing, holding basic cutlery, drawing, and throwing a ball, were performed with the right upper limb. The introduction of swimming training necessitated alternating movements—in order to swim straight, the swimmer must use greater force in the non-dominant limb. This limb was weaker and had a higher fat mass before the study, which is why it required increased training, and the changes are more visible. Similar results of fat mass reduction were presented by Oja et al. in their study [30]. The generalized effect that occurred in swimming may be due to the need to use both the lower and upper limbs to stay afloat in the water. There are no conclusive studies in the literature on the effects of swimming on body composition in children aged 7–10 years. Charmas and Gromisz show in their study that, despite the introduction of swimming training in young females, no changes in body weight and body composition were observed, which the authors explain by limiting the study to physical activity and by not monitoring the diet being followed [38].

Comparing the results of the other two exercise groups, namely taekwondo and tag rugby, one would assume that the observed changes would be similar, as both forms take place in the gymnasium. However, differences in body composition changes were observed from the study.

In the taekwondo training group, the fat mass of the upper limbs decreased, which may have been due to the increased intensity of the exercises. Of course, the lower limbs are responsible for maintaining balance and standing during training, but the intensity of the leg muscles is not sufficient to change body composition. Similar results were obtained by Rutkowski et al. for children training in karate [13]. Different results were obtained for the group practicing rugby tag. This may be due to the training methodology, which in the initial phase emphasizes movement on the field, the speed of the starting reaction, the ability to change direction while running and the orientation in space necessary to evade opponents. Viewed from this perspective, it is easier to understand why changes were observed primarily in the lower limbs in this study group, although they are not statistically significant. There is a lack of studies in the literature covering rugby tag for children, so a comparison can be made between this group and other team games such as football or classic rugby. Seabra et al. in their study observed a reduction in fat mass in overweight and obese boys training football for at least 6 months [39]. Similar results, although on an older age group (12–17 years), were obtained by Vasconcellos et al. with 12 weeks of training [40]. However, these studies were based on overall body composition without differentiation into segments. In addition, the training sessions were conducted at an increased intensity as they targeted individuals with above-normal body weight. In contrast, in children playing contact rugby, there was an increase in fat mass in the torso and right upper limb, which may be due to the nature of the training, which involves lower endurance and fat-reducing elements [41].

In the conducted studies, the assessment of body composition and its distribution was a supplementary element and was assessed solely as a result of exercise. However, the observed changes are worth noting, as these results provide a basis for further research on the impact of exercise on fat mass distribution. The results also demonstrate the importance of incorporating physical activity, even just twice a week, into early school-age children. The greatest changes were observed in the non-dominant upper limb, where fat mass was highest before training. It can be argued that, in conjunction with the improvement in physical fitness, a reduction in fat mass was also achieved and the development of muscle mass essential for further physical development was achieved.

The distribution of fat and muscle mass is important not only for physical fitness but also for proper posture development. Asymmetrical muscle mass distribution can affect posture. Weakness on one side, especially in the lower limbs, can lead to asymmetric pelvic positioning, which, if left uncorrected, can lead to lateral curvature of the spine and rotation, i.e., scoliosis. Weakness of the shoulder girdle, i.e., high fat mass and low muscle mass in the upper limbs, can cause shoulder protraction, deepening cervical lordosis, and forward head posture, which in turn leads to nonspecific neck pain or text neck syndrome.

In the study described, the best results after the training program were achieved by children practicing taekwondo. This group showed statistically significant improvement in all Eurofit test samples. The training format is a key element in taekwondo training. Classes are held on a different surface than standard everyday training, requiring greater balance and concentration. Movements performed during classes involve significant acceleration and local resistance, such as striking a glove, kicking a training dummy, or kicking a Thai bag. This is consistent with studies demonstrating the positive impact of martial arts training on speed, agility, strength, flexibility, coordination, balance, and cardiorespiratory fitness [42]. The tag rugby group demonstrated the greatest improvement in balance, which may be due to the nature of the training described earlier. Throughout the training, the children studied performed alternating starts and stops, sudden changes in direction, and leaning while running to avoid intercepting the ball. At the same time, the participants participated in ball games, focusing on maintaining body posture; this may explain the improved balance observed in this group. Correlation analysis showed that age and body mass index (BMI) correlated positively with balance, which may suggest that older children performed better in trials requiring postural control. This relationship is supported by studies showing that children with higher body weight may show greater static stability, albeit at the expense of motor dynamics [43]. In addition, weak but significant correlations were found between the change in left lower limb fat mass and parameters of explosive strength, flexibility and arm muscle strength, which may indicate a reciprocal relationship between body composition and neuromuscular function [44]. The research presented has shown that any form of physical activity is better than no physical activity. However, it should be borne in mind that each form has specific requirements and can therefore produce different bodily responses. Therefore, in schools, especially as part of initial teaching, children should be introduced to different sports in a playful or introductory form, such as combat sports, swimming, skating, rollerblading or tag rugby, as the teaching standards at school level would encourage children to take up physical activity.

### Limitations

A limitation of the study is the age of the participants, as 7–10 years is a period of intense somatic and motor development. During this time, natural changes in body composition occur, such as an increase in muscle mass, a decrease in relative adiposity and improvements in physical performance resulting from the maturation of the neuromuscular system [45]. These changes can occur independently of the training intervention used, making it difficult to interpret the impact of targeted physical activity unequivocally.

Additionally, the slight differences in anthropometric values between the groups before the intervention may have slightly influenced the results. However, it is already certain that children who do not participate in additional physical activity classes are larger and heavier. The survey should be supplemented with questions about the reasons for not engaging in extracurricular physical activity by the control group.

## 5. Conclusions

In summary, a three-month intervention based on targeted physical activity in the studied children had a beneficial effect on some aspects of body composition, with particular emphasis on the fat component and physical fitness determined on the basis of selected Eurofit tests.

The results suggest that physical activity aimed at developing various motor skills such as balance, flexibility, speed, and coordination should be an element of preventive health care for children.

Further studies with a longer follow-up period and diet monitoring are needed

## Figures and Tables

**Table 1:** Anthropometric values of the subjects before the implementation of targeted physical activity.

Anthropometric Values					Statistical Significance					
	Group IA (n = 20) Median (Q1-Q3)	Group IB (n = 42) Median (Q1-Q3)	Group IC (n = 20) Median (Q1-Q3)	Group II (n = 68) Median (Q1-Q3)	Gr IA vs. Gr IB	Gr IA vs. Gr IC	Gr IA vs. Gr II	Gr IB vs. Gr IC	Gr IB vs. Gr II	Gr IC vs. Gr II
Age [years]	7.89 (6.62 -8.83)	7.63 (7.40 -7.81)	7.12 (7.04 -7.25)	8.10 (7.39 -8.80)	1.000	0.006	0.880	0.002	0.108	0.000
Body height [cm]	131.50 (125.00 -129.00)	129.00 (124.00 -132.00)	129.00 (124.50 -132.00)	132.50 (126.00 -138.00)	1.000	1.000	1.000	1.000	0.079	0.234
Body weight [kg]	26.90 (24.60 -30.60)	26.90 (23.90 -30.60)	24.95 (22.70 -26.70)	33.80 (26.85 -37.10)	1.000	0.557	0.204	0.559	0.008	0.000
BMI [kg/m^2^]	15.90 (15.20 -17.40)	16.55 (15.00 -17.80)	14.45 (14.10 -16.40)	18.80 (17.15 -20.25)	1.000	0.595	0.023	0.163	0.003	0.000
FM%	20.90 (18.70 -23.60)	20.90 (18.60 -24.20)	19.15 (15.15 -20.95)	24.40 (22.25 -27.75)	1.000	0.006	0.880	0.670	0.005	0.000
RL FM%	28.70 (24.40 -31.10)	29.35 (27.30 -32.40)	27.75 (24.40 -29.75)	33.00 (31.25 -36.20)	1.000	1.000	0.000	0.393	0.002	0.000
LL FM%	29.20 (25.00 -31.80)	30.30 (27.40 -32.80)	27.85 (24.30 -29.95)	33.05 (31.45 -36.10)	1.000	1.000	0.007	0.471	0.004	0.000
RA FM%	32.50 (28.00 -37.10)	31.95 (29.30 -36.20)	30.85 (26.15 -32.05)	34.30 (30.05 -37.50)	1.000	0.977	1.000	1.000	0.287	0.025
LA FM%	32.70 (29.20 -39.10)	31.55 (29.20 -37.00)	30.60 (26.20 -33.05)	35.20 (31.10 -38.80)	1.000	0.592	1.000	0.898	0.126	0.005
TR FM%	14.70 (12.00 -18.40)	15.00 (11.80 -18.50)	12.75 (8.80 -14.30)	18.05 (14.95 -20.95)	1.000	0.931	0.161	0.875	0.007	0.000

FM%—fat mass in percentage. RL—right leg; LL—left leg; RA—right arm, LA—left arm; TR—torso.

**Table 2:** Fitness test results before implementation of targeted physical activity.

Fitness Tests					Statistical Significance					
	Group IA (n = 20) Median (Q1-Q3)	Group IB (n = 42) Median (Q1-Q3)	Group IC (n = 20) Median (Q1-Q3)	Group II (n = 68) Median (Q1-Q3)	Group IA vs. Group IB	Group IA vs. Group IC	Group IA vs. Group II	Group IB vs. Group IC	Group IB vs. Group II	Group IC vs. Group II
Balance	1.00 (1.00 -1.00)	50.00 (48.00 -56.00)	42.00 (35.50 -46.50)	49.00 (46.00 -56.00)	0.001	0.395	0.001	0.007	1.000	0.003
Speed of hand movement	50.00 (31.00 -57.00)	52.00 (45.00 -59.00)		46.00 (33.30 -53.00)	1.000		1.000		0.011	
Flexibility	48.00 (34.00 -52.00)	50.00 (44.00 -57.00)	48.00 (43.50 -50.00)	46.00 (31.00 -50.00)	0.518	1.000	1.000	1.000	0.021	1.000
Explosive strength	56.00 (50.00 -65.00)	51.00 (44.00 -56.00)	55.00 (49.00 -56.50)	42.50 (34.50 -50.00)	0.945	1.000	0.000	1.000	0.003	0.001
Dynamic trunk strength	42.00 (29.00 -51.00)	48.00 (41.00 -56.00)		45.00 (33.00 -52.00)	0.232		1.000		0.236	
Upper limb muscular endurance	61.00 (55.00 -68.00)	51.00 (43.00 -62.00)		43.00 (33.30 -50.00)	0.124		0.000		0.001	

**Table 3:** Differences in anthropometric values and fitness tests before and after implementation of targeted physical activity.

Anthropometric Values and Fitness Tests					Statistical Significance					
	Group IA (n = 20) Median (Q1-Q3)	Group IB (n = 42) Median (Q1-Q3)	Group IC (n = 20) Median (Q1-Q3)	Group II (n = 68) Median (Q1-Q3)	Group IA vs. Group IB	Group IA vs. Group IC	Group IA vs. Group II	Group IB vs. Group IC	Group IB vs. Group II	Group IC vs. Group II
BMI 1−2	0.00 (−0.40 -0.50)	0.15 (−0.30 -0.50)	0.20 (−0.15 -0.40)	−0.10 (−0.55 -0.30)	1.000	1.000	1.000	1.000	0.540	1.000
FM% 1−2	−0.40 (−0.90 -1.50)	0.55 (−0.80 -1.40)	−0.80 * (−1.95 -0.50)	0.10 (−1.05 -1.05)	1.000	1.000	1.000	0.234	1.000	0.895
RL FM% 1−2	−0.80 (−1.50 -0.20)	0.90 (−0.70 -2.00)	0.05 (−1.00 -1.05)	0.65 * (−0.70 -1.65)	0.081	1.000	0.101	1.000	1.000	1.000
LL FM% 1−2	−0.60 (−1.60 -1.40)	0.85 (−0.60 -1.70)	0.25 (−0.75 -0.75)	0.45 (−0.95 -1.25)	0.754	1.000	1.000	1.000	1.000	1.000
RA FM% 1−2	0.10 (−1.70 -2.30)	0.60 (−0.70 -2.50)	−1.95 * (−3.25 -0.40)	0.15 (−1.10 -1.55)	1.000	0.481	1.000	0.039	1.000	0.115
LA FM% 1−2	1.20 (−1.00 -2.20)	0.80 * (−0.60 -2.00)	−2.20 * (−3.35 -0.45)	−0.20 (−1.80 -1.55)	1.000	0.061	1.000	0.027	0.878	0.335
TR FM% 1−2	−0.30 (−0.90 -1.50)	0.50 (−0.80 -2.00)	−1.30 * (−2.70 -1.00)	−0.20 (−1.20 -1.15)	1.000	0.749	1.000	0.090	1.000	0.423
Balance 1−2			−11.00 (−20.00 -7.00)	0.00 (−4.00 -9.00)						0.000
Speed of hand movement 1−2	−8.00 * (−27.00 -3.00)	3.00 (−2.00 -47.00)	0.00 (0.00 -0.00)	0.00 (−11.00 -7.50)	0.000	0.051	0.077	0.765	0.011	1.000
Flexibility 1−2	−3.00 (−11.00 -0.00)	28.00 (0.00 -53.00)	4.50 (−1.50 -16.50)	3.00 (−1.00 -16.67)	0.000	0.065	0.033	0.477	0.021	1.000
Explosive strength 1−2	−4.00 * (−12.00 -1.00)	13.50 (−1.00 -51.00)	0.00 (−7.50 -7.50)	0.00 (−7.00 -10.50)	0.001	0.713	0.608	0.313	0.013	1.000
Dynamic trunk strength 1−2	−5.00 * (−10.00 -1.00)	3.50 (−1.00 -43.00)	0.00 (0.00 -0.00)	0.00 (−6.00 -7.50)	0.000	0.072	0.008	0.427	0.105	1.000
Upper limb muscular endurance 1−2	−7.00 * (−12.00 -1.00)	8.00 (0.00 -57.00)	0.00 (0.00 -0.00)	15.83 (−4.00 -43.00)	0.000	0.309	0.001	0.032	0.203	1.000

* Statistically significant difference *p* < 0.05.

**Table 4:** Relationships between changes in anthropometric data and changes in physical fitness.

	Age	BMI 1	FM% 1−2	RL FM% 1−2	LL FM% 1−2	RA FM% 1−2	LA FM% 1−2	TR FM% 1−2
Balance 1−2	0.441	0.490	−0.068	0.015	−0.016	0.071	−0.040	−0.081
Speed of hand movement 1−2	0.048	−0.043	0.058	0.197	0.156	0.079	−0.054	0.022
Flexibility 1−2	−0.077	−0.155	0.091	0.193	0.124	0.073	0.035	0.035
Explosive strength 1−2	−0.052	−0.074	0.113	0.224	0.172	0.071	0.026	0.073
Dynamic trunk strength 1−2	−0.033	0.006	−0.013	0.155	0.134	−0.048	−0.094	−0.053
Upper limb muscular endurance 1−2	0.177	−0.025	0.010	0.184	0.142	0.100	−0.046	−0.026

## Data Availability

The data that support the findings of this study are available from the corresponding author upon request. The data used in this manuscript are part of a large research project, at the same time they are data of children and for the protection of personal data they are only available on request from the corresponding author.
